# Functional near-infrared spectroscopy during optic flow with and without fixation

**DOI:** 10.1371/journal.pone.0193710

**Published:** 2018-03-07

**Authors:** Carrie W. Hoppes, Patrick J. Sparto, Susan L. Whitney, Joseph M. Furman, Theodore J. Huppert

**Affiliations:** 1 Department of Physical Therapy, University of Pittsburgh, Pittsburgh, Pennsylvania, United States of America; 2 Department of Otolaryngology, University of Pittsburgh, Pittsburgh, Pennsylvania, United States of America; 3 Department of Radiology, University of Pittsburgh, Pittsburgh, Pennsylvania, United States of America; Tokai University, JAPAN

## Abstract

**Background and purpose:**

Individuals with visual vertigo describe symptoms of dizziness, disorientation, and/or impaired balance in environments with conflicting visual and vestibular information or complex visual stimuli. Physical therapists often prescribe habituation exercises using optic flow to treat these symptoms, but there are no evidence-based guidelines for delivering optic flow and it is unclear how the brain processes such stimuli. The purposes of this study were to use functional near-infrared spectroscopy (fNIRS) to explore cerebral activation during optic flow, and determine if visual fixation had a modulating effect on brain activity.

**Methods:**

Fifteen healthy participants (7 males and 8 females; mean age 41 years old) stood in a virtual reality environment and viewed optic flow moving unidirectionally in the yaw plane with and without fixation. Changes in cerebral activation were recorded from the bilateral fronto-temporo-parietal and occipital lobes using fNIRS.

**Results:**

Cerebral activation was greater with visual motion than while viewing a stationary scene. Greater cerebral activation in the bilateral fronto-temporo-parietal lobes was observed when optic flow was viewed with fixation.

**Discussion and conclusions:**

Optic flow activates the bilateral fronto-temporo-parietal regions of the cerebral cortex. This activation is greater while viewing optic flow and a fixation target, providing preliminary evidence supporting the use of a fixation target during habituation exercises.

## Introduction

Visual vertigo describes symptoms of dizziness, disorientation, and/or impaired balance induced by environments with conflicting visual and vestibular information or complex visual stimuli.[1] Individuals with vestibular disorders often report exacerbation of their symptoms in such environments, which can lead to avoidance behaviors resulting in activity limitations and participation restrictions.[2] Additionally, these visually-dependent individuals may display increased postural sway with full-field visual motion[1, 3] which may place them at greater risk for falling.

A clinical practice guideline on vestibular rehabilitation for peripheral vestibular hypofunction recommends habituation exercises as a treatment approach when busy visual environments exacerbate dizziness.[4] These habituation exercises may involve optokinetic stimuli or virtual reality environments,[4–7] and have been shown to decrease visual vertigo symptoms when incorporated into rehabilitation regimens.[8, 9] While optokinetic stimuli are often utilized by clinicians, evidence-based stimulus parameters are not yet known and its mechanisms are poorly understood.

Optokinetic stimuli are a subtype of optic flow, which is the continual change of images on the retina that occurs from movement of the visual environment. Studies using positron emission tomography (PET) [10] and functional magnetic resonance imaging (fMRI) [11] during optokinetic stimuli viewed with fixation reveal a reciprocal visual-vestibular inhibitory pattern. Activation of the visual cortex co-occurs with deactivation of the parietoinsular vestibular cortex. This pattern may reflect sensory re-weighting of visual and vestibular afferent information, in which the more reliable or dominant sensory input is given greater weight.[10] It is not known if individuals with visual vertigo respond to optokinetic stimuli in the same manner, or if fixation modulates this relationship.

Optokinetic stimuli can induce a feeling of self-motion in a stationary observer known as vection. Vection is important for the judgement, control and guidance of self-motion,[12] and essential for spatial orientation, locomotion, and navigation. A synthesis of the relevant literature indicates that fixation enhances the perception of vection. This may be explained by the increased optic flow afforded by suppression of optokinetic nystagmus which causes the visual stimuli to repeatedly move across the retina.[13] There are some discrepancies in the literature regarding the influence of fixation on perception of vection, however, with Stern et al. reporting that fixation decreased ratings of vection.[14] Fixation suppression of optokinetic nystagmus resulted in increased activation in the supplementary eye field and anterior cingulate gyrus, unchanged activation in the visual cortex, decreased activation in most of the ocular motor areas, and suppressed activation in the anterior and posterior insula and the thalamus.[15] Given these observed changes in brain activation in healthy individuals during fixation suppression, the presence or absence of a fixation target during vestibular rehabilitation may have important clinical implications.

A better understanding of the normal mechanisms of cortical processing of optic flow information is a necessary first step in establishing optimal rehabilitation regimens that include habituation exercises. There is no evidence to support or refute the use of a fixation target during habituation to optic flow. Whether or not a patient is asked to fixate on a target during progressive exposure to optic flow is at the discretion of the treating physical therapist, which leads to variation in practice patterns and patient outcomes. Evidence for the use of a fixation target during treatment using optic flow would help to standardize delivery of optic flow as an intervention for individuals with visual vertigo.

Different neuroimaging modalities have been used to study cortical regions related to the integration and reweighting of visual, vestibular, and somatosensory afferent information. In particular, imaging of the vestibular cortex has included fMRI, PET, magnetoencephalography, electroencephalography, and functional near-infrared spectroscopy (fNIRS). fMRI has been used in conjunction with caloric testing to identify the vestibular cortex as the temporoparietal junction and posterior insula.[16] In response to galvanic vestibular stimulation, Bense et al. found vestibular activation (anterior insula, paramedian and dorsolateral thalamus, putamen, inferior parietal lobule, precentral and middle frontal gyrus, middle and superior temporal gyrus, and the anterior cingulate gyrus, in addition to the bilateral cerebellar hemispheres), but noted deactivation in the visual cortex.[17] This is evidence of a reciprocal visual-vestibular interaction that lends support to sensory re-weighting. To further explore this visual-vestibular interaction, the bilateral fronto-temporo-parietal and occipital lobes were selected as regions of interest for this study. The regions of interest overlie the middle frontal gyrus, superior temporal gyrus, and the extrastriate visual cortical regions and were selected because of their relevance to visual and vestibular stimulation reported in previous studies.[17–23]

Understanding the mechanisms of cortical processing of optic flow information was previously limited to restrictive neuroimaging techniques. These included studies using PET[10, 24–26] and fMRI[15, 21, 27–29] that require the participant to lie supine during image acquisition. fNIRS has emerged as a neuroimaging modality that allows for upright imaging of the patient during functional tasks such as balance and gait. fNIRS has be used to measure changes in cerebral activation during a simple balance task [30], during balancing on a balance board,[31] in response to perturbations [32], while walking [33, 34], and while running [34]. fNIRS allows for study of reweighting of visual, vestibular, and somatosensory afferent information as real-time postural challenges are induced during optic flow. Use of fNIRS to study optic flow has been extremely limited thus far. Wijeakumar et al.[35] explored cortical activation in the primary visual cortex in response to a moving visual stimulus. Their study was not designed to assess cortical activation in the middle temporal region, the area of the brain that responds preferentially to optic flow in humans. Participants were also studied in a presumably seated position.

This study was performed to better understand the processing of optic flow information in the upright observer and the relationship between cortical activation and fixation. Study of this relationship was conducted by examining changes in cerebral activation using fNIRS in healthy adults exposed to constant velocity optic flow with and without a fixation target.

## Materials and methods

### Participants

Twenty-three healthy participants were screened, and fifteen participants (7 males and 8 females; mean age 41 years old, range 20–67; 13 right-handed, 1 left-handed, and 1 ambidextrous) were included in the study. One individual who was eligible to participate withdrew prior to the experimental visits due to a change in health status, and seven individuals were excluded based on at least one of the following exclusion criteria: (a) a history of otologic or neurologic disease; (b) history of migraine; (c) corrected binocular vision worse than 20/40, macular degeneration, or glaucoma; (d) results of screening tests that indicated vestibular asymmetry or loss; (e) history of excessive motion sickness; (f) medication use that may affect balance; (g) unwillingness to abstain from alcohol for 48 hours prior to testing; (h) known pregnancy; and/or (i) body mass greater than 118 kilograms. Written informed consent was obtained from all participants and the study was approved by the University of Pittsburgh Institutional Review Board.

### Design

This was a cross-sectional study with two experimental visits approximately one week apart. During the first visit, participants viewed optic flow stimuli with a fixation target and during the second visit, participants viewed the same stimuli without fixation. An alternating-condition blocked design (e.g., A-B-A-B-A) was employed, where A was comprised of a stationary visual field and B was comprised of a moving visual field. During B, optic flow in the yaw plane was moving either randomly or unidirectionally (leftward or rightward) at a constant velocity. Four five-minute trials were conducted at each of the visits. The order of these trials was randomly assigned, such that the participant would view two trials that were randomly moving and two trials that were moving unidirectionally.

### Visual stimuli

**Fig 1:**
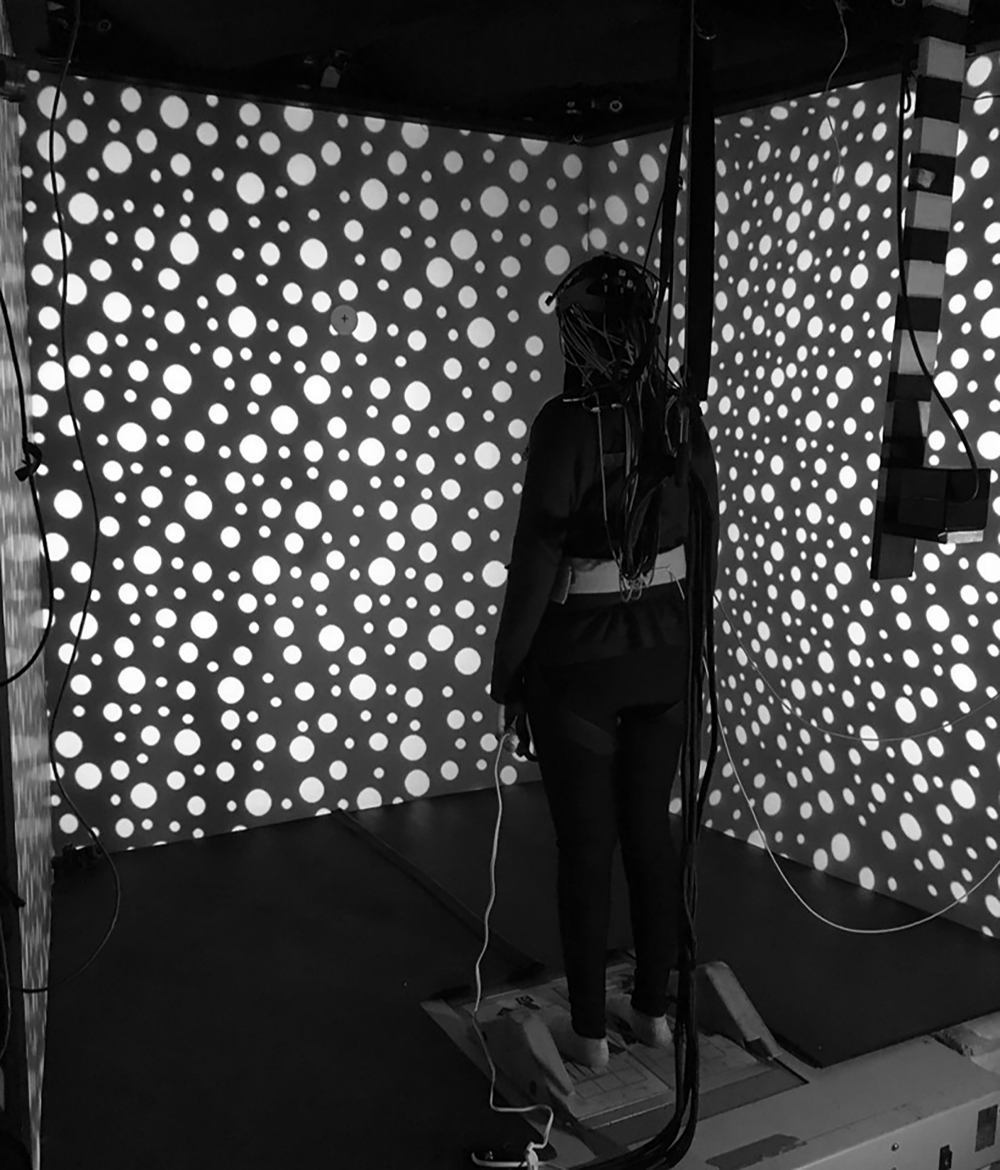
Virtual reality environment. The participant was exposed to optic flow in a three-screen wide field-of-view virtual reality environment. Participants faced the front screen that was 1.5 meters away, and were tethered to the ceiling to prevent falls.

The visual stimulus consisted of 720 white dots randomly placed on a black background. The dots were of three sizes (3.4, 2.5, and 1.5 degrees of visual arc) and covered approximately 20% of the total area. The baseline condition was stationary. For the optic flow conditions, the visual field moved either randomly (sum of four sines: frequencies = π/31, π/11, π/7, π/5 Hz; at an RMS velocity of 40 degrees per second) or unidirectionally at a constant velocity of 40 degrees per second in the yaw plane. The randomly moving optic flow was intended to be a control condition that did not induce self-motion (vection); however, the randomly moving visual field did induce vection in some participants (median = 1, range 1–5; see Motion Sickness Assessment Questionnaire below) and was not further analyzed since it did not produce the intended experimental condition. The visual stimulus was back-projected onto a three-screen wide field-of-view (180 degrees horizontal and 70 degrees vertical) (Fig 1).

### Measurements

#### Vection: Motion Sickness Assessment Questionnaire

It was important to measure vection because activation in the middle temporal visual areas (MT+ and V6), ventral intraparietal area, and parietoinsular vestibular cortex is greater during the experience of self-motion.[29] The Motion Sickness Assessment Questionnaire (MSAQ) was developed and validated to assess motion sickness as a multidimensional construct.[36] As there is no consistent use of rating scales for quantifying vection in the published literature, item 14 (“I felt like I was spinning”) of the MSAQ rated on a one (“not at all”) to nine (“severely”) scale was used as a measure of vection intensity. The MSAQ was administered at baseline, following each of the four trials, and at the conclusion of the experimental visit.

#### Cortical activation: Near-infrared spectroscopy

fNIRS is a non-invasive functional neuroimaging method that measures changes in the volume and oxygenation of the blood. The change in intensity of visible red to near-infrared light between sources and detectors that are placed on the scalp is measured. During imaging, flexible fiber optic cables deliver low levels of light (<0.4 W/cm^2^) to the sources on the scalp. This light diffuses through the tissues to a depth of approximately 5–8 mm in the outer cerebral cortex.[37] Light that is not absorbed is detected and flexible fiber optic cables carry the light back to photon detectors within the fNIRS instrument. During brain activity, there are regional changes in oxyhemoglobin (HbO_2_) and deoxyhemoglobin (Hb) concentration that change the absorption of light. As a result of neurovascular coupling, increased HbO_2_ and decreased Hb is typically observed for functional responses.[38]

**Fig 2:**
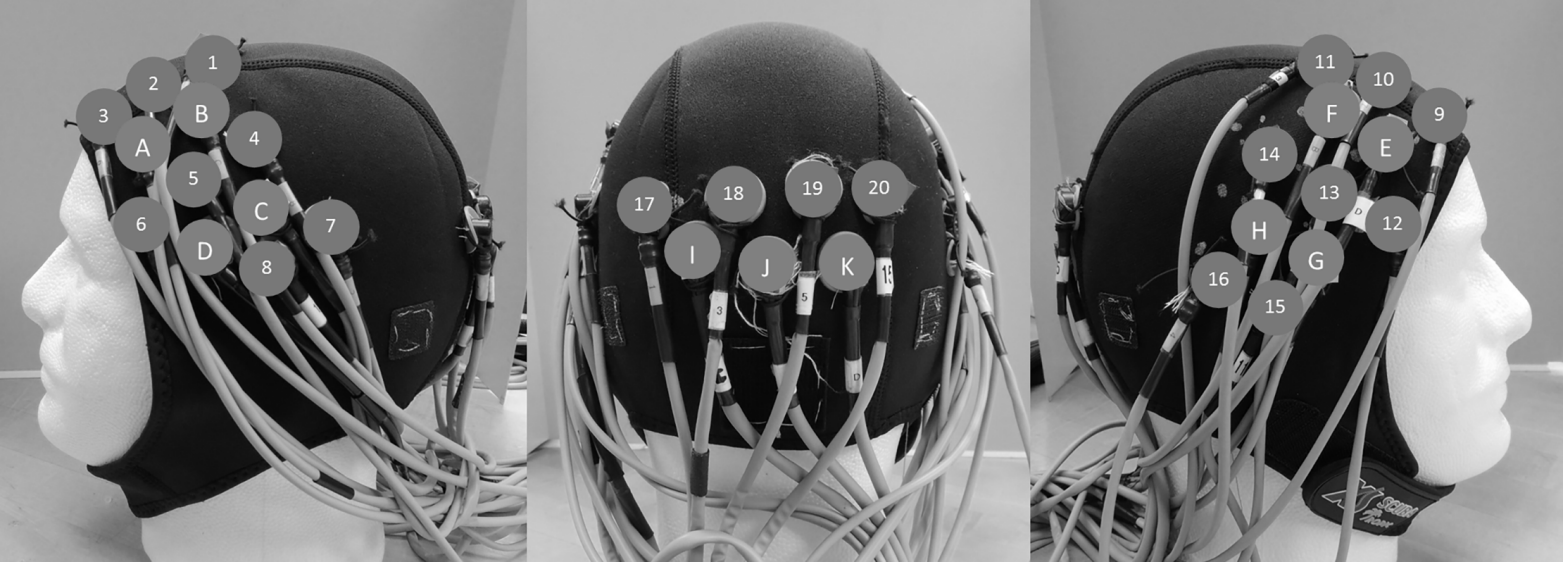
fNIRS head cap. The participant wore a fNIRS head cap that consisted of 11 sources (capital letters A-K) and 20 detectors (numbers 1–20) on the scalp, distributed between the left fronto-temporo-parietal (left), occipital (center), and right fronto-temporo-parietal (right) regions.

A 32-channel continuous wave fNIRS instrument (CW6 Real-time system; TechEn, Inc.; Milford, MA) was used to record changes in HbO_2_ and Hb concentration at 830 nm and 690 nm, respectively.[39, 40] A fNIRS head cap consisting of 11 sources and 20 detectors located over the bilateral fronto-temporo-parietal and occipital lobes was used (Fig 2) to assess multisensory vestibular (temporoparietal region, supramarginal gyrus, and supratemporal gyrus) and extrastriate visual (V2, V3, V3A) brain regions. Optical data from 72 source-detector combinations (36 source-detector combinations at two wavelengths) was collected at 20 Hz and down sampled to 4 Hz using a custom-built MATLAB-based acquisition software program.[41]

fNIRS data are recorded as changes in the light from a source position incident on a detector position as a function of time. Signals are first converted to changes in optical density over time and then converted to HbO_2_ and Hb estimates, as a measure of cortical activity, via the modified Beer-Lambert law with a partial path length correction of 0.1 for both wavelengths.[40]

### Statistical analysis

Demographic and MSAQ data were analyzed using IBM SPSS Statistics 22 (IBM Corporation, Armonk, NY) and optical data were analyzed using MATLAB (Mathworks, Natick, MA). The time-course of hemoglobin changes for each source-detector pair was analyzed using a general linear model (GLM) Δ[Hbx]=X*β+ϵ where X is the design matrix encoding the timing of stimulus events and β is the coefficient (weight) of that stimulus condition for that source-detector channel. The design matrix was constructed from the convolution of the stimulus timing and duration with a canonical response model (see details in [41]).

In this analysis, no preprocessing was applied. Instead, physiological noise and motion artifacts were dealt with statistically within the GLM.[42] To reduce effects of motion artifacts and systemic physiology, an iteratively auto-regressively whitened, weighted least-squares model was used to solve the general linear equation.[41] This regression model uses an n^th^ order auto-regressive filter determined by an Akaike model-order selection to whiten both sides of the GLM expression. The regression coefficients (β) and their error-covariance (Covβ) are estimated, and used to define statistical tests between task conditions or baseline. The subject-level analysis to investigate if the optic flow stimulus elicited a significant brain activation compared with the stationary visual field was done using a GLM with a boxcar function of the timing of the optic flow stimulus as a regressor.[41] The timing of the A-B-A-B-A design was specified in the design matrix. The regression model is solved sequentially for each data file for each participant. All source-detector pairs within a file are solved concurrently yielding a full covariance model of the noise, which is used in group-level analysis. T-tests were used to determine if the regression coefficients were statistically non-zero.

Group-level analysis was performed using a linear mixed effects model, using the task-related regression weights (β) from the first-level GLM as the dependent variable and subject as a random effect. A modified version of the MATLAB function fitLME (linear mixed effects model estimator) was used to solve the weighted maximum likelihood estimate of the parameters. The model was whitened using the error-covariance (Covβ) of the first level GLM model. To control for multiple comparisons, a false discovery rate(FDR)-correction was used with the significance level set at 0.05 (q ≤ 0.05).[43] In this study, group-level analysis to examine the effect of fixation versus no fixation was done using a linear mixed effects model that looked at the fixation condition as the fixed effect and subject as a random effect. Three regions of interest were defined (left and right fronto-temporo-parietal and occipital). Each of the fronto-temporo-parietal regions of interest, comprised of four sources and eight detectors, had fifteen channels. The occipital region of interest, comprised of three sources and four detectors, had six channels.

Descriptive statistics were calculated for demographic variables and MSAQ ratings. The MSAQ ratings during the two exposures to optic flow were averaged. A Wilcoxon signed rank test was used to explore differences in MSAQ ratings between fixation and no fixation conditions, as a Shapiro-Wilk test revealed the MSAQ ratings were not normally distributed.

## Results

### Vection

A Wilcoxon signed rank test indicated that ratings of vection intensity were not different between fixation (*M* = 2.55, *SD* = 2.00) and no fixation (*M* = 2.50, *SD* = 1.66) conditions (*Z* = -.182, *p* = .855).

### Cortical activation

Statistical analyses revealed significant activation in the three regions of interest when viewing optic flow with a fixation target compared to a stationary visual field (Table 1). Increased HbO_2_ concentration was seen in all three regions of interest: left fronto-temporal-parietal (T = 9.41, *p* < 0.0001, FDR-corrected), right fronto-temporal-parietal (T = 5.55, *p* < 0.0001, FDR-corrected), and occipital (T = 2.55, *p* = 0.015, FDR-corrected). These were coupled with decreased Hb concentration in all three regions of interest: left fronto-temporal-parietal (T = -6.97, *p* < 0.0001, FDR-corrected), right fronto-temporal-parietal (T = -9.24, *p* < 0.0001, FDR-corrected), and occipital (T = -6.62, *p* < 0.0001, FDR-corrected). A similar trend was seen without fixation, however, decreased Hb concentration in the left fronto-temporo-parietal lobe (T = -0.96, *p* = 0.338, FDR-corrected) and decreased HbO_2_ concentration in the right fronto-temporo-parietal lobe (T = -1.85, *p* = 0.070, FDR-corrected) were not statistically significant.

**Table 1:** Change in cerebral activation when viewing optic flow compared to a stationary visual field.

Visit	Region of Interest	Chromophore	Beta	SE	T	*p*	*q*
Fixation	Left fronto-temporo-parietal	HbO_2_	1.447	0.154	9.41	< 0.0001	< 0.0001*
		Hb	-0.618	0.089	-6.97	< 0.0001	< 0.0001*
	Right fronto-temporo-parietal	HbO_2_	0.800	0.144	5.55	< 0.0001	< 0.0001*
		Hb	-0.756	0.082	-9.24	< 0.0001	< 0.0001*
	Occipital	HbO_2_	0.848	0.333	2.55	0.0110	0.0147*
		Hb	-1.063	0.160	-6.62	< 0.0001	< 0.0001*
No Fixation	Left fronto-temporo-parietal	HbO_2_	0.575	0.157	3.65	0.0003	0.0004*
		Hb	-0.093	0.097	-0.96	0.3380	0.3380
	Right fronto-temporo-parietal	HbO_2_	-0.309	0.167	-1.85	0.0642	0.0700
		Hb	-0.211	0.097	-2.18	0.0294	0.0352*
	Occipital	HbO_2_	1.383	0.301	4.59	< 0.0001	< 0.0001*
		Hb	0.370	0.141	2.62	0.0089	0.0133*

Beta = regression coefficients; DF = degrees of freedom; HbO_2_ = oxyhemoglobin; Hb = deoxyhemoglobin; *p* = *p*-value; *q* = *q*-value; SE = Standard Error; T = T-statistic

* indicates *p* ≤ 0.05, false discovery rate-corrected

**Fig 3:**
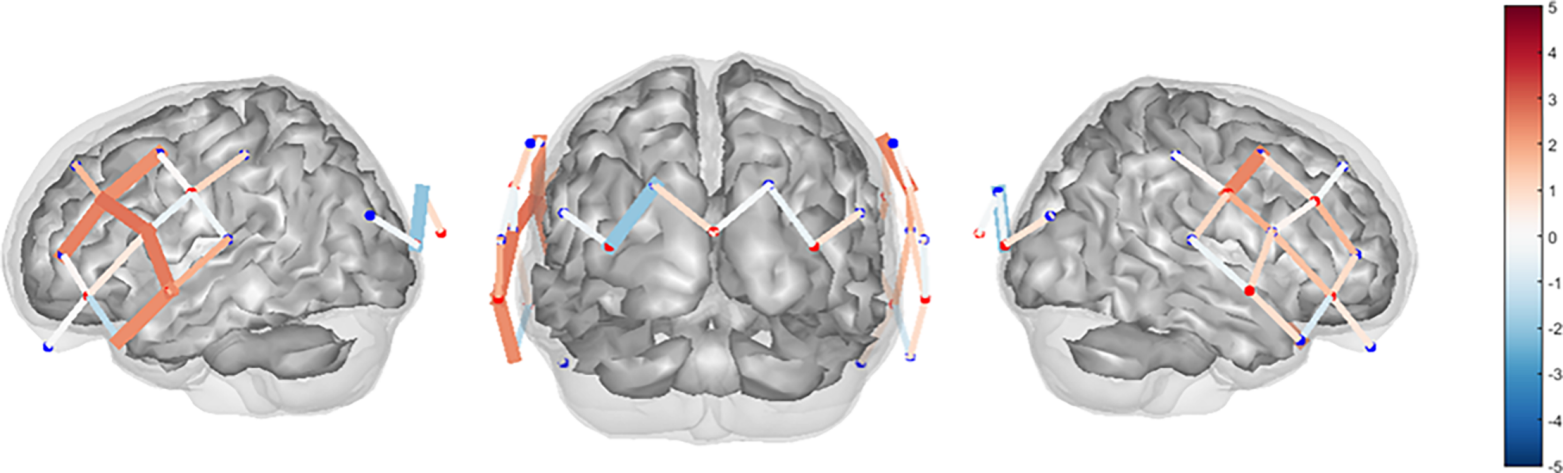
Estimated spatial maps. Estimated spatial maps (T-test) of the oxyhemoglobin data collected using fNIRS for the change in cerebral activation between fixation and no fixation conditions using all source-detector combinations. The color bar represents the results of the T-statistic (T-score). Areas in red indicate greater cerebral activation (increased oxyhemoglobin) and areas in blue indicate lesser cerebral activation (decreased oxyhemoglobin) during the comparison. Thick lines indicate areas with significant activation (*p* < 0.05).

Statistical analyses using a linear mixed effects model to explore fixation versus no fixation revealed significant activation in the bilateral fronto-temporo-parietal lobes (Table 2). Increased HbO_2_ and decreased Hb concentration were noted in the left fronto-temporo-parietal lobe. A similar trend was seen in the right fronto-temporo-parietal lobe, however, the decreased Hb concentration was not statistically significant (T = -0.92, *p* = .426, FDR-corrected). Decreased Hb concentration was also noted in the occipital lobe with fixation compared to without fixation, though changes in HbO_2_ concentration were not significant (T = -0.36, *p* = .725, FDR-corrected). Estimated spatial maps (T-test) of the HbO_2_ data for the change in cerebral activation between fixation and no fixation conditions using all source-detector combinations for the three regions of interest are shown in Fig 3.

**Table 2:** Change in cerebral activation when viewing optic flow moving unidirectionally in the yaw plane with a fixation target compared to without a fixation target.

Region of Interest	Chromophore	Beta	SE	T	*p*	*q*
Left fronto-temporo-parietal	HbO_2_	7.465	2.377	3.14	0.0016	0.0051*
	Hb	-8.769	2.026	-4.33	< 0.0001	< 0.0001*
Right fronto-temporo-parietal	HbO_2_	8.414	2.970	2.83	0.0046	0.0092*
	Hb	-2.216	2.396	-0.92	0.3550	0.4260
Occipital	HbO_2_	-0.468	1.329	-0.35	0.7247	0.7247
	Hb	-2.957	1.087	-2.72	0.0065	0.0098*

Beta = regression coefficients; HbO_2_ = oxyhemoglobin; Hb = deoxyhemoglobin; *p* = *p*-value; *q* = *q*-value; SE = Standard Error; T = T-statistic;

* indicates *p* ≤ 0.05, false discovery rate-corrected

## Discussion

This study was performed to better understand the processing of optic flow information when viewing optic flow in comparison to a stationary visual field, with and without a fixation target. Study of this relationship was conducted by examining changes in cerebral activation using fNIRS, which revealed greater activation when viewing optic flow in comparison to a stationary visual field. Additionally, greater activation was seen in the bilateral fronto-temporo-parietal lobes when optic flow moving unidirectionally in the yaw plane was viewed with a fixation target.

When viewing optic flow with a fixation target compared to a stationary visual field, the expected pattern of increased HbO2 and decreased Hb was observed. This general pattern was also observed when viewing optic flow without a fixation target compared to a stationary visual field, though decreased Hb concentration in the left fronto-temporo-parietal lobe was not statistically significant. Decreased HbO2 concentration in the right fronto-temporo-parietal lobe when viewing optic flow without a fixation target compared to a stationary visual field was also not statistically significant. Perhaps, without a fixation target, the optic flow was not sufficient to produce vection requiring additional vestibular related activity in the right superior temporal region to stabilize posture.

When viewing optic flow with a fixation target compared to without a fixation target, increased HbO_2_ and decreased Hb concentration were noted in the left fronto-temporo-parietal lobe. A similar trend was seen in the right fronto-temporo-parietal lobe, however, the decreased Hb concentration was not statistically significant. Decreased Hb concentration was also noted in the occipital lobe with fixation compared to without fixation, though changes in HbO2 concentration were not significant. This decreased HbO2 concentration in the occipital regions of interest might represent deactivation of the extrastriate visual cortex. Previous studies using PET[10] and fMRI[11] during optokinetic stimuli viewed with fixation revealed a reciprocal visual-vestibular inhibitory pattern, with activation of the visual cortex co-occurring with deactivation of the parietoinsular vestibular cortex. We observed activation of the fronto-temporo-parietal regions and deactivation of the occipital regions, which may relate to the upright task. Participants in this study were imaged while standing, and this may account for the reversal of the pattern of reciprocal inhibition observed during PET and fMRI studies where subjects were imaged in supine. Subjects in this study may require additional vestibular related activity in the bilateral superior temporal regions to stabilize posture.

The mechanisms of cortical processing of optic flow information was previously limited to restrictive neuroimaging techniques. fMRI during yaw or pitch optokinetic stimulation revealed that a fixation target suppressed optokinetic nystagmus and resulted in increased activation in the supplementary eye field and anterior cingulate gyrus, unchanged activation in the visual cortex, decreased activation in most of the ocular motor areas, and suppressed activation in the anterior and posterior insula and the thalamus.[15] The anterior and posterior insula are deep within the lateral sulcus (underneath the fronto-temporo-parietal regions of interest in this study), and cannot be imaged with fNIRS. During unidirectional optic flow in the yaw plane viewed with a fixation target, there was increased activation in the bilateral fronto-temporo-parietal lobes in the region of the supramarginal gyrus. This region is involved in sensory reweighting of visual, vestibular, and proprioceptive system information. Similar to this response to optic flow during quiet stance, fNIRS revealed increased activation in the superior temporal gyrus and supramarginal gyrus at the temporoparietal junction during a video game skiing task.[30] There was no difference in HbO_2_ concentration in the occipital lobe, which may be explained by the presence of optic flow in both viewing conditions. The larger decrease in Hb signals in the occipital lobe during the no fixation condition is unclear at this time.

To date, no other studies have used fNIRS to explore optic flow and the perception of vection. Fixation is known to enhance the perception of vection. This may be explained by suppression of optokinetic nystagmus, which causes the visual stimuli to repeatedly move across the retina.[13] This may lead to greater afferent input for central processing in cortical regions responsive to visual motion and optic flow information. Visual stimuli appear to move faster when gaze is fixated on the static (black) background, than when smooth pursuit is used to follow the dots.[13] Previous research has found that participants perceived greater estimates of vection with higher velocity visual motion.[44] Our findings indicate that the presence of a fixation target during unidirectionally moving optic flow designed to induce vection increased cortical activation. Fixation may have suppressed the optokinetic nystagmus in our participants, leading to greater cortical processing of visual motion and optic flow information as indicated by increased HbO_2_ concentration in the bilateral fronto-temporo-parietal lobes in comparison to viewing optic flow without fixation. Previous research found that a fixation target during linear left to right optic flow decreased vection latencies and increased vection duration.[13] Decreased vection latencies were also found with fixation compared to without fixation during yaw and pitch optic flow.[45]

The MSAQ was originally developed to assess motion sickness,[36] but was used in this study to quantify vection. In this sample, no difference was found in subjective ratings of vection intensity using the MSAQ when the optic flow stimulus was viewed with and without a fixation target. This suggests that item 14 of the MSAQ may not be sensitive to changes in perception of vection intensity. The observed changes in HbO_2_ and Hb concentration when the optic flow stimulus was viewed with and without a fixation target may indicate that fNIRS could provide an objective measure of vection intensity to complement subjective ratings captured using self-report. Palmisano et al. discuss the challenges of conducting vection research, to include the difficulties of using self-reported, subjective ratings of vection.[12] Vection can be challenging for the naïve participant to understand and accurately report, so physiological indicators like eye movements, cortical and postural responses may provide further support for confirming the subjective experience of self-motion.[12]

Although participants stood in a wide field-of-view, some participants reported that they could see the ceiling. This would provide a visual reference that may have decreased ratings of vection intensity. Though subjective ratings of vection intensity were not different, cerebral activation was different with and without a fixation target. This may indicate that the optic flow information was subconsciously perceived differently than verbally reported.

There were several limitations to this study. First, this was a pilot study using a small sample of healthy participants, so the study findings do not yet translate to clinical populations. It is also not known if greater activity during fixation is beneficial. Second, limited areas were imaged with fNIRS due to the head cap design and depth of penetration. These areas, however, overlie the middle frontal gyrus, superior temporal gyrus, and the extrastriate visual cortical regions and were selected because of their relevance to visual and vestibular stimulation reported in previous studies.[17–23] The middle frontal region spanned an area from the superior frontal gyrus to the inferior frontal gyrus, and superior temporal region spanned an area from the inferior frontal gyrus to the middle temporal gyrus. The regions of interest, therefore, adequately covered those areas of the cortex most relevant to the research questions. An additional limitation of this study was that the presentation of the visual stimuli was not counterbalanced for fixation and no fixation conditions. This may have resulted in an order effect. Attention is known to affect the strength of vection.[46] Although participants viewed four trials with fixation before four trials without fixation, data collection occurred on different days so fatigue and/or attention were likely mitigated.

## Conclusions

While optokinetic stimuli are often utilized by physical therapists during vestibular rehabilitation therapy, evidence-based stimulus parameters are not yet known. This work provides a beginning understanding of the mechanisms of cortical processing of optic flow information in the upright observer, which is a necessary first step in establishing optimal rehabilitation regimens. Physical therapists progress patients during vestibular rehabilitation by performing habituation exercises in increasingly more functional positions like standing. Unlike other neuroimaging modalities, fNIRS allowed for study of processing of optic flow information in the upright observer under conditions that replicate delivery of habituation exercises using optokinetic stimuli or virtual reality environments in the clinic. Greater cortical activation in the bilateral fronto-temporo-parietal lobes provides preliminary support for the use of a fixation target during habituation to optic flow. It is not yet known if this holds true for individuals with complaints of visual vertigo, and warrants further investigation in this population.
